# Aboriginal Families Study: a population-based study keeping community and policy goals in mind right from the start

**DOI:** 10.1186/1475-9276-12-41

**Published:** 2013-06-14

**Authors:** Mary Buckskin, Jackie Ah Kit, Karen Glover, Amanda Mitchell, Roxanne Miller, Donna Weetra, Jan Wiebe, Jane S Yelland, Jonathan Newbury, Jeffrey Robinson, Stephanie J Brown

**Affiliations:** 1 Aboriginal Health Council of South Australia, 9 King William Road, Unley, South Australia 5061, Australia; 2 Women’s and Children’s Health Network, 295 South Terrace, Adelaide, South Australia 5000, Australia; 3 Pangula Mannamurna Inc, 191 Commercial Road West, Mt Gambier, South Australia 5290, Australia; 4 Healthy Mothers Healthy Families Research Group, Murdoch Childrens Research Institute, Flemington Road, Parkville, VIC 3052, Australia; 5 Discipline of Rural Health, The University of Adelaide, PO Box 3200, Port Lincoln, South Australia 5005, Australia; 6 Discipline of Obstetrics and Gynaecology, The University of Adelaide, Adelaide, South Australia 5005, Australia; 7 School of Population and Global Health, The University of Melbourne, Parkville, VIC 3052, Australia

**Keywords:** Antenatal care, Health inequalities, Indigenous health, Maternal health, Participatory research, Perinatal health outcomes

## Abstract

**Background:**

Australian Aboriginal and Torres Strait Islander women are between two to five times more likely to die in childbirth than non-Aboriginal women, and two to three times more likely to have a low birthweight infant. Babies with a low birthweight are more likely to have chronic health problems in adult life. Currently, there is limited research evidence regarding effective interventions to inform new initiatives to strengthen antenatal care for Aboriginal families.

**Method/Design:**

The Aboriginal Families Study is a cross sectional population-based study investigating the views and experiences of Aboriginal and non-Aboriginal women having an Aboriginal baby in the state of South Australia over a 2-year period. The primary aims are to compare the experiences and views of women attending standard models of antenatal care with those accessing care via Aboriginal Family Birthing Program services which include Aboriginal Maternal Infant Care (AMIC) Workers as members of the clinical team; to assess factors associated with early and continuing engagement with antenatal care; and to use the information to inform strengthening of services for Aboriginal families. Women living in urban, regional and remote areas of South Australia have been invited to take part in the study by completing a structured interview or, if preferred, a self-administered questionnaire, when their baby is between 4–12 months old.

**Discussion:**

Having a baby is an important life event in all families and in all cultures. How supported women feel during pregnancy, how women and families are welcomed by services, how safe they feel coming in to hospitals to give birth, and what happens to families during a hospital stay and in the early months after the birth of a new baby are important social determinants of maternal, newborn and child health outcomes. The Aboriginal Families Study builds on consultation with Aboriginal communities across South Australia. The project has been implemented with guidance from an Aboriginal Advisory Group keeping community and policy goals in mind right from the start. The results of the study will provide a unique resource to inform quality improvement and strengthening of services for Aboriginal families.

## Introduction

Australian Aboriginal and Torres Strait Islander women are between two to five times more likely to die in childbirth than non-Indigenous women, and two to three times more likely to have a low birthweight infant [1]. Babies with a low birthweight are more likely to die in infancy [2], more likely to be admitted to neonatal intensive care [3], and may be more likely to have serious health problems (e.g. cardiovascular disease, diabetes) in adult life [4]. Recent data suggest that in some Australian states, including South Australia, the proportion of low birthweight babies born to Aboriginal mothers may be increasing [5,6]. The Australian Government has set agreed targets for closing the gap in Indigenous disadvantage outlined by the Council of Australian Governments (COAG) in the National Indigenous Reform Agenda [7]. Under the terms of this agreement, federal, state and territory governments have committed to closing the gap in life expectancy between Aboriginal and non-Aboriginal Australians within a generation, and halving the gap in mortality rates for Aboriginal children under five within a decade. Key performance indicators for the National Indigenous Reform Agenda include: an increase in the proportion of Aboriginal and Torres Strait Islander mothers receiving antenatal care in the first trimester of pregnancy (≤13 weeks’ gestation) and in the proportion of Aboriginal and Torres Strait Islander mothers attending five or more antenatal visits; and a reduction in the proportion of Aboriginal and Torres Strait Islander infants with a low birthweight (<2,500 grams).

New funding made available under the COAG National Partnership Agreement on Indigenous Early Childhood Development has facilitated a range of new programs and initiatives to strengthen antenatal care and child and maternal health services for Aboriginal families in all Australian states and territories [8]. Currently there is a dearth of research evidence regarding effective intervention strategies to inform these initiatives [9-12]. Most of the available evidence comes from small-scale local evaluation studies, predominantly undertaken in regional and remote locations [13-20]. The roll out of COAG funding under the National Partnership Agreement has in effect created an Australia wide ‘natural experiment’ in seeking to improve maternal and perinatal outcomes for Aboriginal and Torres Strait Islander women and children. It is vital that lessons learned from the range of programs being developed and implemented with COAG funding are captured by concurrent evaluation at a state and territory level. However, there is still no complete national perinatal data for Aboriginal mothers and babies. State and territory based perinatal data collections vary in their capacity to ascertain Aboriginal and Torres Strait Islander status of mothers and infants, and steps have only recently been taken to include information regarding status of the infant in the minimum data set for most state jurisdictions [21].

This paper describes the development of a statewide study in South Australia that aims to invite approximately 300 women giving birth to an Aboriginal baby to talk about their experiences of using services during pregnancy, labour and birth, and the first 4–12 months after having a baby. The study has been developed by researchers based at the Murdoch Childrens Research Institute and the University of Adelaide, in partnership with the Aboriginal Health Council of South Australia Inc. (AHCSA). The project arose in the context of planning for a population-based postal survey of recent mothers in South Australia and Victoria. In 2006, the researchers planning this survey approached the AHCSA about working in partnership on a project to provide avenues for Aboriginal women’s voices to be included in the research. At our initial meeting we discussed the idea of seeking funding to facilitate consultations with Aboriginal community organisations and communities in South Australia as a way to seek input into development of the research. The Aboriginal Families Study is the name given to the project that grew out of these discussions. South Australia where the project is based is one of six Australian states, and covers a geographic area four times the size of the UK.

Often when researchers approach Aboriginal community organisations and/or communities, they already have a fairly well developed research question and study protocol in mind. We did not. This paper charts the social history of the project, and outlines the steps we took to get from our initial discussions in 2006 to the stage of developing the study protocol, governance arrangements, and procedures for carrying out the study. These include: obtaining ‘in principle’ support from the Board of Management of the AHCSA for the conduct of consultations with Aboriginal communities about the project; development of a project agreement between MCRI and the AHCSA; establishment of an Aboriginal Advisory Group to guide the consultations, and subsequently, the development of the study protocol, and conduct of the research; statewide consultations with Aboriginal communities, policy makers and service providers preceding development of the study design and methods; a lengthy pilot study phase that tested different versions of the study questionnaire and recruitment procedures; obtaining ethics approval from a variety of institutional ethics committees; development of a Research Agreement covering governance arrangements for the research phase of the study signed by all partner organisations and study investigators; appointment and training of the fieldwork team; through to recruitment and interviewing of women in urban, regional and remote areas of South Australia.

## Methods

### Establishing partnerships and governance arrangements for community consultation

In May 2006, three members of the research team (SB, JY, GS) were invited to attend a meeting of the Board of Management of the AHCSA in Whyalla to discuss our proposal to seek funding for consultations with Aboriginal communities in South Australia about the development of a research project. Whyalla is 380 kilometres north west of Adelaide, and has a relatively large Aboriginal population. At the Board Meeting, we were asked why we wanted to do the project, who would own the information gathered in the course of the research, and what would come out of it for Aboriginal communities. Most of all Board Members wanted to know “Will it make a difference?”. Key messages that emerged from discussions with the Board included: the importance of focusing on the whole family; of taking into consideration social factors that influence health and well-being; the need for communities to have a say in whether or not the project should go ahead; and above all, that research would only be welcomed by Aboriginal communities if people could see ways in which it would lead to better services and outcomes for Aboriginal families.

After this meeting, the AHCSA Board gave ‘in principle’ support for us to proceed with an application to the National Health and Medical Research Council (NHMRC) for seed funding to undertake a 12 month consultation in South Australia. The next steps in formalising collaboration were taken in mid-2007 after seed funding (as part of a larger application to conduct a population based survey of women giving birth in South Australia and Victoria) was secured. This involved developing a project agreement between MCRI and the AHCSA (signed in September 2007) and establishing an Aboriginal Advisory Group to guide the conduct of consultations and development of the research. The Aboriginal Advisory Group - comprising representatives from metropolitan and regional health services, the AHCSA, Aboriginal Elders’ Council and Aboriginal Health Workers with expertise in maternity and postnatal care - has met regularly, approximately 6–8 times a year, to provide advice and direction to the research team.

### Community and key stakeholder consultations

Two part-time Aboriginal research officers – one based in Adelaide and the other in Port Lincoln on the West Coast of Spencer Gulf - facilitated community consultations in urban, regional and remote communities in South Australia over a 15-month period (October 2007-December 2008). Consultations were held in Adelaide and the major regional centres of Port Augusta, Port Lincoln, Whyalla, West Coast, mid North and Yorke Peninsula, Ceduna, Coober Pedy, Yalata, Point Pearce and Mt Gambier. In addition, consultations were held with policy makers and service providers in a range of metropolitan and regional settings. Recurring themes throughout the consultations were: the importance of family, social context, and social health issues to women’s health and wellbeing during pregnancy; the impact on women and families of needing to travel and stay away from home in order to attend regional and/or metropolitan health services; the impact of seeing many different non-Aboriginal health professionals throughout pregnancy, birth and the postnatal period; and lack of information about local community health services for women and families with a new baby. The consultations demonstrated support for the research to go ahead provided that it was community-led and directed towards improving pregnancy, birthing and postnatal services for Aboriginal families. Two reports documenting findings from the consultation were produced in early 2009: a full report and a community report for providing feedback to communities taking part [22,23]. Both are available via the project website [24].

### Obtaining approval for research questions and study methods

The major research questions to be addressed in the study, and overall design and methods for the Aboriginal Families Study were developed between mid 2008 and early 2009 drawing on findings from community and key stakeholder consultations, and the advice of the Aboriginal Advisory Group. The study protocol - including aims, methods, and governance arrangements for the study - was approved by the Board of the AHCSA in June 2009, providing the basis for development and pilot testing of data collection methods over the next 12 months.

### Policy context and formation of the Aboriginal Families Study Policy Implementation Group

Coinciding with this stage of development of the study, the Council of Australian Governments (COAG) announced funding for new initiatives to strengthen antenatal care and maternal and child health services for Aboriginal communities in all states and territories. In South Australia, COAG funding has been used to roll out an Aboriginal Family Birthing Program (AFBP) based on a model that had been in operation in Port Augusta and Whyalla since 2004. The program enables Aboriginal women to be cared for during pregnancy, labour and birth and the postnatal period by Aboriginal Maternal and Infant Care (AMIC) Workers working in partnership with midwives and doctors [25]. Since 2009, Local Health Networks covering metropolitan and regional areas of South Australia have been working to expand the Aboriginal Family Birthing Program across urban, regional and remote communities in South Australia.

In December 2008, the research team, together with members of the Aboriginal Advisory Group, invited senior policy makers in the South Australian Health Department, the Women’s and Children’s Health Network and Country Health SA to a meeting to discuss the relevance of the Aboriginal Families Study to current policy directions in South Australia. As an outcome of this meeting, the decision was taken to establish a formal partnership between the AHCSA, SA Health and the research institutions involved in the study, initially with the aim of submitting a joint funding application to NHMRC. This application submitted in early 2009 was unsuccessful, but the organisations and individuals that were party to this application agreed to continue working together to secure funding and facilitate translation of research findings. Funding for the study was secured via an NHMRC project grant (#1004395) awarded in 2010, and grants from the Rio Tinto Aboriginal Fund and SA Health.

### Aims and hypotheses

The major aims of the study are to:

1. Investigate the views and experiences of a population-based sample of Aboriginal women and women with an Aboriginal partner having a baby in South Australia (i.e. mothers of Aboriginal babies) regarding pregnancy, birthing and postnatal services;

2. Compare the experiences of women attending standard (‘mainstream’) models of public antenatal care (e.g. public clinic care, shared care) with those of women accessing antenatal care via a co-ordinated program receiving support from COAG under the National Partnership Agreement on Indigenous Early Childhood Development and involving clinical care from a multidisciplinary team including Aboriginal Maternal Infant Care Workers (Aboriginal Family Birthing Program);

3. Assess factors associated with early and continued engagement of Aboriginal families with antenatal care;

4. Compare the experiences of women taking part in the Aboriginal Families Study with the experiences of non-Aboriginal women taking part in a population-based survey of women giving birth in South Australia;

5. Use information gathered in the study to inform early intervention strategies and appropriate care pathways for Aboriginal women and families, especially those experiencing psychological distress and/or social health issues during and after pregnancy;

6. Build capacity for collaborative Aboriginal health research addressing the needs of Aboriginal women and families with young children.

We hypothesised that compared to mothers of Aboriginal babies attending standard or ‘mainstream’ public models of antenatal care (e.g. public hospital antenatal clinic care, shared care between a public hospital and community based general practitioner), mothers of Aboriginal babies who attend Aboriginal Family Birthing Program services will be *more likely* to have their first antenatal visit in the first trimester of pregnancy (≤13 weeks’ gestation) and to attend five or more antenatal visits. In addition, we hypothesised that mothers of Aboriginal babies who attend Aboriginal Family Birthing Program services will be *more likely* to receive support in relation to social health issues; and to report positive experiences of antenatal, intrapartum and postnatal care; and less likely to report experiences of being treated unfairly or discriminated against by health professionals.

### Study population

All women giving birth to an Aboriginal baby in South Australia between July 2011 and June 2013 excluding women under 14 years of age were eligible to take part in the study. Women who give birth interstate (e.g. at Alice Springs Hospital), but normally resided in South Australia were also eligible to participate. Women eligible to take part have been invited to participate in an interview when their baby is approximately six months of age (range 4–12 months) consistent with the timing of the *2008 SA Healthy Mothers Healthy Families Survey*[26,27].

### Sample size

Data collected by the South Australian Pregnancy Outcome Unit show that over 600 Aboriginal women give birth in South Australia each year [5,6]. Around 60% of Aboriginal women in South Australia give birth in metropolitan public hospitals, some travelling from regional areas. Data on paternity are not recorded in the South Australian Pregnancy Outcome dataset so it is not possible to use these data to identify the number of non-Aboriginal women with an Aboriginal partner giving birth in South Australia. Australian Bureau of Statistics data on births in South Australia indicate that there were 976 Aboriginal births registered in 2008 [28], but it is likely that there is some misclassification and under-ascertainment of Aboriginal births in these data [29]. Based on the available figures we estimated the total number of women eligible to participate in the study over a 2-year period to be approximately 2000, with around half living outside the Adelaide metropolitan area. Given that 53% of births to Aboriginal mothers in South Australia are to women aged less than 25 years [5,6], and more than half live outside the metropolitan area, particular attention has been given to strategies to recruit younger women aged 14 to 24 years and women living in regional and remote areas.

Power calculations were conducted at the beginning of the study to assess the required sample size for testing study hypotheses. Since the proportion of Aboriginal mothers receiving antenatal care in the first trimester, and proportion of Aboriginal mothers attending five or more antenatal visits are national targets for health system performance [7,8], we estimated required sample size based on these outcomes. We assumed a ratio of 2:1 for women attending mainstream public antenatal care versus women attending Aboriginal Family Birthing Program services. Based on this assumption, a sample of 330 women with a sub-group size of 80 women attending AFBP services and 160 women attending mainstream public antenatal care with alpha of 0.05 will provide 80% power to detect: i) a 20% absolute increase in the proportion of women attending a first antenatal visit at ≤13 weeks’ gestation (from 41% in standard or ‘mainstream’ models of public antenatal care to 61% in AFBP services) and (ii) a 15% increase in the proportion of women attending 5 or more visits (from 75% in standard models of public antenatal care to 90% in AFBP services).

### Recruitment and conduct of interviews

Recruitment strategies include: invitation via public maternity hospitals, primary care and other local services; promotion of the study through community events, posters, leaflets and the Aboriginal media; and drawing on contacts and relationships formed during the extensive period of community consultation. By May 2013, over 350 women had expressed interest in taking part in the study, and 200 women had completed the study questionnaire placing the study on track to achieve a final sample size of approximately 300 participants by December 2013. Preliminary analyses of the first 130 participants show that 18% (23/130) were aged 15–19 years, and 36% 20–24 years (47/130), matching the expected age distribution for births to Aboriginal women in South Australia [6]. Fifty-eight percent of the first 130 participants gave birth at a metropolitan teaching hospital, 40% at a regional hospital, 2% at home or on the way to hospital, and less than 2% in a private hospital, matching the expected distribution for place of birth based on routinely collected perinatal data for South Australia [6]. While more than half of the first 130 participants gave birth in a metropolitan hospital, 56% resided outside the major metropolitan city of Adelaide. Included in the first 130 participants are women living in urban, regional and remote areas from across South Australia, representing more than 20 Aboriginal language and community groups.

There are four main methods via which women have been recruited to the study: 1) an interviewer visiting women while they are in hospital after the birth of their baby and inviting them to register with the study; 2) a health service or other agency informing women about the study and asking them to agree to their contact details being passed on to the research team; 3) an interviewer talking to women at community events, and 4) women hearing about the study from women who have already completed a study questionnaire, and agreeing to their contact details being passed on to an interviewer. Women expressing interest in the study have been followed up by phone, and arrangements are then made for an interviewer to meet with them to provide more information about the study prior to seeking consent to participation. All women interested in taking part have been given an information package about the study, including a Participant Information Sheet, which is also explained by the interviewer before seeking written or oral consent. Young women aged 14–17 years have been encouraged to discuss the information sheet with a parent or guardian, but do not need parental or guardian consent in order to participate.

Interviews following a structured interview schedule have been undertaken by a team of Aboriginal research interviewers: three based in Adelaide, and five in regional centres, including Port Lincoln, Port Augusta and Murray Bridge. Interviews have been conducted in a range of community settings (e.g. early childhood services, Aboriginal health services) as well as in women’s homes. If preferred, women may also opt to have a non-Aboriginal interviewer or to complete the ‘interview schedule’ as a self-administered questionnaire. Study participants are given a supermarket gift voucher to thank them for taking part. All interviewers have participated in training specifically developed for the study, with ongoing training and support provided by the Fieldwork Co-ordinator (DW) and other senior members of the research team (JW, RM, SB). Detailed protocols for recruitment of women to the study, seeking and obtaining informed consent, and conduct of interviews are documented in the *Aboriginal Families Study Interviewer Guidelines*. These guidelines also cover health and safety considerations for interviewers working in urban, regional and remote locations.

### Data collection

Table 1 provides an overview of data collected in the Aboriginal Families Study questionnaire. A pilot study undertaken in 2010 established the acceptability and feasibility of using a structured interview schedule, and allowed for refinement of interview questions. Several versions of the interview schedule were piloted with women living in Adelaide and in regional and remote communities. Feedback on the questionnaire was also sought from service providers, policy makers and from members of the Aboriginal Advisory Group. A copy of the questionnaire will be made available on request to the corresponding author. Researchers and/or organisations wishing to utilise the questionnaire (or components of the questionnaire) in other research contexts are requested to contact the corresponding author to seek written approval.

**Table 1:** Data collected in the Aboriginal Families Study questionnaire*

	**Mother**	**Baby**
**Social characteristics**	Date of birth	Date of birth
	Aboriginality	Aboriginality
	Aboriginal language group/community	Number of siblings
	Place of residence (metro/regional/remote)	
	Number and age of other children	
	Education	
	Employment	
	Health care concession card	
	Access to transport	
**Antenatal care**	Gestation at first antenatal check-up	
	Number of antenatal check-ups	
	Model of care (e.g. public clinic, Aboriginal Family Birthing Program)	
	Location of antenatal care (e.g. hospital, home)	
	Hospital admission during pregnancy	
	Required to travel and be away overnight in order to access tests or specialist level care	
**Birth events**	Hospital admission prior to onset of labour	Place of birth
	Intrapartum transfer to another hospital	Infant birthweight
	Caregivers present during labour/birth	Gestational age
	Family present during labour/birth	
	Method of birth	
**Postnatal care**	Length of postnatal hospital stay	Admission to Neonatal Intensive Care or Special Care Nursery
	Home visits after discharge	
	Contacts with primary care services	Initiation and duration of breastfeeding
	Support in relation to infant feeding	
**Women’s views of care**	Access to information	
	Involvement in decisions about care	
	Satisfaction with pain relief	
	Interaction with health professionals	
	Perceived discrimination (e.g. talked down to, stereotyped, treated unfairly)	
	Support provided if needed to travel/be away from home for care during pregnancy and/or to give birth	
**Social health issues**	Social health issues (e.g. housing problems, legal issues, drug and alcohol problem, family violence)	
	Smoking during and after pregnancy	
**Health and well being**	Medical conditions during pregnancy	
	Postpartum physical health problems	
	Postpartum psychological distress	

* Copy of the questionnaire available on request to the corresponding author.

All women taking part in the study have been asked to give consent for record linkage to routinely collected perinatal and child health data sets, and permission for future follow-up. Data from consenting participants will be linked with routinely collected health data from a range of population databases including data collected by the South Australian Pregnancy Outcome Unit (perinatal data) and SA Health (Child Health Record). Record linkage will be facilitated by SA-NT Data Link [30]. Additional funding is being sought currently to follow-up a sub-sample of families to invite them to participate in a study focusing on childhood resilience. This planned follow-up study takes up a community priority identified in the community consultations conducted at the outset of the Aboriginal Families Study.

### Ethics approval and funding

Ethics approval for the study was first of all obtained from the Aboriginal Health Research Ethics Committee (AHREC) of the Aboriginal Health Council of South Australia. Institutional ethics approval has also been obtained from SA Health, Women’s and Children’s Health Network, Lyell McEwin Hospital, the Royal Children’s Hospital and the University of Adelaide.

### Building capacity for collaborative Aboriginal health research

A major objective of the Aboriginal Families Study is to build capacity for collaborative ‘community-led’ Aboriginal health research. Establishing agreed governance arrangements for the research phase of the study has been an important tool for clarifying roles and expectations of partner organisations and study investigators, and underpins the way that the Aboriginal Families Study collaborators are working together to conduct the study. Governance arrangements are defined in a Research Agreement developed over a series of meetings in 2010–2011, and signed by all partner organisations and study investigators in early 2012. This agreement covers: roles and responsibilities of partner organisations and study investigators; ethics clearance and reporting; Aboriginal cultural and intellectual property rights; storage, access and archiving of research materials; analysis and interpretation of results; and publication and dissemination of research findings (including acknowledgements and authorship). The agreement recognises the obligations of study investigators named on the National Health and Medical Research Council (NHMRC) project grant awarded to the study in 2011, but places this within the broader context of the agreement between the parties to conduct the study in accord with the *NHMRC Values and Ethics*: *Guidelines for Ethical Conduct in Aboriginal and Torres Strait Islander Health Research and RoadMap II frameworks *[31,32]. In practice, this means that the study investigators and research team report to the Aboriginal Advisory Group, which in turn reports to the Board of the Aboriginal Health Council of South Australia. An Executive Committee comprising the CEO of the AHCSA (MB), the Chairperson of the Aboriginal Advisory Group (KG) and the Principal Investigator (SB) was established to act as an out of session source of advice and support for the Principal Investigator, and is also responsible for providing advice regarding key decisions relating to the progress of the study between meetings of the Aboriginal Advisory Group.

The study is also providing opportunities for building capacity and research skills for Aboriginal health research within the team of researchers working together to conduct the study. Across the team participating in the conduct of the research there is a wealth of community knowledge and expertise, and connections to communities across South Australia. Three senior members of the fieldwork team have completed the Certificate IV qualification in Indigenous research capacity building (EA, RM, DW). The Interviewer Guidelines for the study were developed collaboratively over a 12-month period drawing on the collective knowledge and expertise of members of the Aboriginal Advisory Group, Aboriginal members of the fieldwork team, and non-Aboriginal study investigators and fieldwork team members. The training program for research interviewers comprised an initial series of two training blocks, with regular times for the fieldwork team to get together to share knowledge and review how things are going at roughly two month intervals throughout the fieldwork phase. The AHCSA was involved in planning of the initial training modules and conducted a workshop on research ethics in the early stages of fieldwork. A set of core principles to inform the way we work with each other, and how we work with study participants and study investigators, were developed collaboratively, and approved by the Aboriginal Advisory Group, prior to commencement of the fieldwork phase of the study. In general, presentations at conferences are co-presented by Aboriginal and non-Aboriginal members of the fieldwork team and/or members of the Aboriginal Advisory Group. Procedures for quality assurance, data analysis and interpretation of study findings are being managed collaboratively to maximise opportunities for capacity building and exchange.

## Discussion

The Aboriginal Families Study is underpinned by strong community, policy and research partnerships that have been developed over an extended period of working together to develop the project. As others have argued, this takes time, resources, flexibility and a commitment to ‘mutually respectful partnerships’ [33-35]. In the Aboriginal Families Study, research questions and study methods were defined collaboratively following extensive statewide community consultations with Aboriginal communities and discussions with policy makers. Development of the study protocol and study instruments happened over an extended period, with many opportunities for community input and feedback. The study represents a long-term investment by the AHCSA, MCRI and the University of Adelaide in partnership and collaborative development of ‘community-led’ Aboriginal health research focusing on health system reform in South Australia. Three sections of SA Health – the Women’s and Children’s Health Network, Country Health SA, and the South Australian Department of Health – have also made substantial contributions to the project via involvement in the Aboriginal Families Study Policy Implementation Partnership, and via seed funding awarded to the study prior to securing the NHMRC project grant.

The study findings will provide important information about the experiences of Aboriginal families accessing both mainstream services and new services funded by COAG. In particular, the study will provide avenues for Aboriginal women’s voices about their experiences of using services to be heard by policy makers and service providers with responsibility for quality improvement and strengthening of the current round of COAG initiatives. Too often when initiatives like these are implemented the people most affected by changes to services do not have a voice in the process. The Aboriginal Families Study aims to ensure that the voices of Aboriginal women and families are accessible to policy makers, health service managers and service providers as evidence to inform ongoing efforts to strengthen services. By keeping community and policy goals in mind right from the start, the project is laying important foundations for sustained improvements in Aboriginal women’s and children’s health.

In addition, the study is providing opportunities for capacity building and capacity exchange through the process of working together to develop and implement the study. This is occurring at all levels of the study, and involves all the major contributors, including Aboriginal and non-Aboriginal researchers, members of the Aboriginal Advisory Group and members of the Policy Implementation Partnership.

Having a baby is an important life event in all families and in all cultures. How supported women feel during pregnancy, how women and families are welcomed by services, how safe they feel coming in to hospitals to give birth, and what happens to families during a hospital stay and in the early months after the birth of a new baby are important social determinants of maternal, newborn and child health outcomes. The Aboriginal Families Study provides a unique resource to inform quality improvement and strengthening of services for Australian Aboriginal women and families in South Australia and nationally. The study is also a testament to what can be achieved by collaboration and partnership.

## Endnotes

The term ‘Aboriginal’ used throughout this paper is intended to refer to people of Aboriginal and Torres Strait Islander origin.

## Abbreviations

AFBP: Aboriginal Family Birthing Program; AHCSA: Aboriginal Health Council of South Australia; AHREC: Aboriginal Health Research Ethics Committee; AMIC: Aboriginal Maternal Infant Care Worker; COAG: Council of Australian Governments; MCRI: Murdoch Childrens Research Institute; NHMRC: National Health and Medical Research Council; SA: South Australia.

## Competing interests

The authors declare that they have no competing interests.

## Authors’ contributions

MB, JAK, and KG contributed to study conception, design and conduct of the study as members of the Aboriginal Advisory Group. RM undertook consultations with Aboriginal communities, and contributed to the conception and design of the study, and piloting of study procedures and study instruments. SB, JY, KG, AM, JN, and JR contributed to study conception, design and conduct of the study. SB, JW and JY co-wrote the successful grant application to NHMRC, and applications for ethical approval, in collaboration with other study investigators. SB, RM, JW, and JY co-facilitated the consultation and pilot stages of the project with guidance from members of the Aboriginal Advisory Group. JW, DW, RM and SB developed the interviewer guidelines and procedures for fieldwork. SB wrote the initial draft of the manuscript. All authors were involved in revising the manuscript and have given final approval for the manuscript to be published.

## Writing group

Mary Buckskin, Jackie Ah Kit, Karen Glover, Amanda Mitchell, Roxanne Miller, Donna Weetra, Jan Wiebe, Jane Yelland, Jonathan Newbury, Jeffrey Robinson, and Stephanie J Brown.

## Chief investigators

Stephanie Brown, Jane Yelland, Karen Glover, Amanda Mitchell, Jonathan Newbury, Jeffrey Robinson, Fiona Arney (Australian Centre for Child Protection, University of South Australia, GPO Box 2471, Adelaide South Australia, 5001), Georgie Stamp (to mid 2012) (Discipline of Rural Health, The University of Adelaide, PO Box 3200, Port Lincoln, South Australia, 5005).

## Fieldwork team (in alphabetic order)

Eunice Aston, Deborah Fuschtei, Jessie King, Rosie King, Emily Jaworek, Karen McCue, Roxanne Miller, Jess Reid, Deelia Richards, Denise Thomas, Donna Weetra, Jan Wiebe, Hayley Wilson, Jannaya Wilson, Rikki Wilson.

## Aboriginal Advisory Group

Karen Glover (Chairperson), Jackie AhKIt, Tania Axleby-Blake, Marilyn Bingapore-Lee, Mary Buckskin, Kathy Chisholm (Co-chair), Glenise Coulthard (Co-chair), Margaret Hampton, Deanna Stuart-Butler, May Turner, Sharron Williams.

## Policy Implementation Partnership

Kathy Chisholm (Chairperson), Fiona Arney, Tania Axleby-Blake, Kerry Bowering, Stephanie Brown, Anna Caponi, Glenise Coulthard, Susan Donath, Lucy Evans, April Lawrie-Smith, Kim Morey, Jonathan Newbury, Pauline McEntee, Amanda Mitchell, Verity Patterson, Heather Petty, Jeffrey Robinson, Trish Strachan, Jane Yelland.
